# Chemical and Genetic Diversity of *Nodularia spumigena* from the Baltic Sea

**DOI:** 10.3390/md14110209

**Published:** 2016-11-10

**Authors:** Hanna Mazur-Marzec, Mireia Bertos-Fortis, Anna Toruńska-Sitarz, Anna Fidor, Catherine Legrand

**Affiliations:** 1 Department of Marine Biotechnology, University of Gdansk, Marszałka J. Piłusudskiego 46, 81378 Gdynia, Poland; biohm@ug.edu.pl (H.M.-M.); anna.torunska@ug.edu.pl (A.T.-S.); anna.fidor77@gmail.com (A.F.); 2 Department of Biology and Environmental Science, Center of Ecology and Evolution in Microbial Model Systems, Linnaeus University, 39182 Kalmar, Sweden; mireia.bertos.fortis@lnu.se

**Keywords:** *Nodularia spumigena*, Baltic Sea, cyanobacteria, chemotaxonomy, non-ribosomal peptides, PC-IGS

## Abstract

*Nodularia spumigena* is a toxic, filamentous cyanobacterium occurring in brackish waters worldwide, yet forms extensive recurrent blooms in the Baltic Sea. *N. spumigena* produces several classes of non-ribosomal peptides (NRPs) that are active against several key metabolic enzymes. Previously, strains from geographically distant regions showed distinct NRP metabolic profiles. In this work, conspecific diversity in *N. spumigena* was studied using chemical and genetic approaches. NRP profiles were determined in 25 *N. spumigena* strains isolated in different years and from different locations in the Baltic Sea using liquid chromatography-tandem mass spectrometry (LC-MS/MS). Genetic diversity was assessed by targeting the phycocyanin intergenic spacer and flanking regions (*cpc*BA-IGS). Overall, 14 spumigins, 5 aeruginosins, 2 pseudaeruginosins, 2 nodularins, 36 anabaenopeptins, and one new cyanopeptolin-like peptide were identified among the strains. Seven anabaenopeptins were new structures; one cyanopeptolin-like peptide was discovered in *N. spumigena* for the first time. Based on NRP profiles and *cpc*BA-IGS sequences, the strains were grouped into two main clusters without apparent influence of year and location, indicating persistent presence of these two subpopulations in the Baltic Sea. This study is a major step in using chemical profiling to explore conspecific diversity with a higher resolution than with a sole genetic approach.

## 1. Introduction

*Nodularia spumigena* is a bloom-forming, filamentous and N_2_-fixing cyanobacterium from the Nostocales order. This species occurs in many brackish waters worldwide, but most extensive blooms are reported in estuaries, lagoons and inland waters of Australia and New Zealand [1,2,3], and the Baltic Sea [4]. These blooms are toxic due to the production of the cyclic pentapeptide nodularin (NOD), and can have negative impacts on ecosystems and water quality [5,6]. In addition to nodularin, *N. spumigena* produces a wide range of chemically diverse non-ribosomal peptides (NRPs) [7]. Due to the high morphological diversity of planktonic *Nodularia* [8,9], four species of this cyanobacterium were distinguished: *N. spumigena*, *N. baltica*, *N. litorea* and *N. crassa* [10]. Based on the analysis of 16S rRNA gene sequences coupled to taxonomic studies, it is currently accepted that only the species *N. spumigena* is present in the Baltic Sea plankton [11]. Within the species, many distinct phenotypes can co-exist with different morphological, biochemical and physiological traits [9,11,12,13,14]. Whether the metabolic diversity of NRPs in *N. spumigena* is related to genetic diversity has not been tested.

*N. spumigena* constitutes a prolific source of bioactive secondary metabolites classified as non-ribosomal peptides (NRPs). Biosynthesis of these compounds proceeds on large multienzyme complexes called non-ribosomal peptide synthetases (NRPS), composed of several modules [15,16]. Some cyanobacterial peptides, such as microcystins and nodularins, are synthesized by a hybrid nonribosomal peptide and polyketide pathway. Polyketide synthases (PKSs) are also composed of modules which catalyze the activation, incorporation and optional modification of the carboxylic acid components of a peptide. The structure of NRPSs and PKSs as well as function of peptides synthesized on these enzyme complexes have been discussed in many review papers [17,18]. It was suggested that the metabolites might perform interchangeable and complementary functions and help cyanobacteria to survive under different biotic and abiotic factors [19].

**Figure 1:**
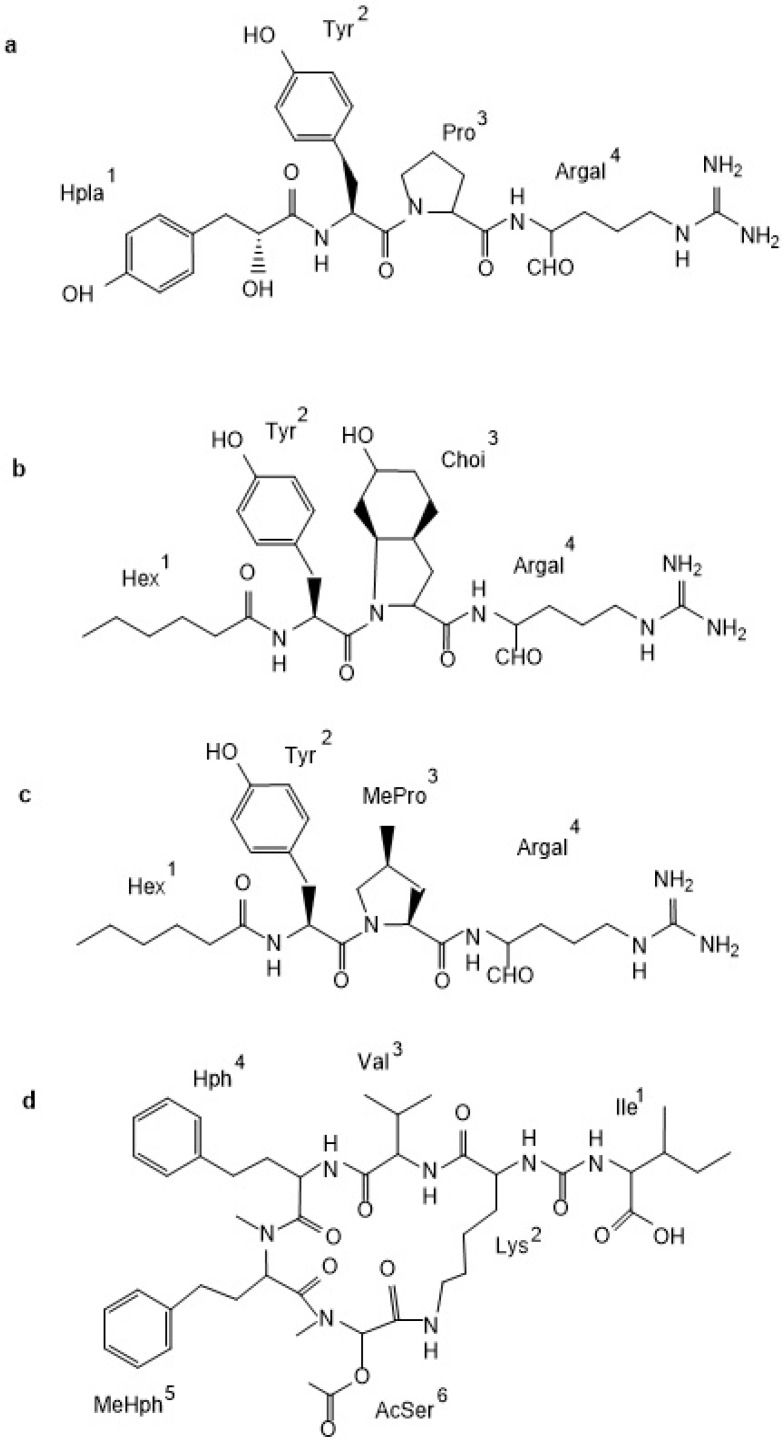
Structure of peptides produced by *Nodularia spumigena* from the Baltic Sea: spumigin (SPU 582a; **a**), aeruginosin (NAL2; **b**), pseudoaeruginosin (NS2; **c**), and nodulapeptin (NP849; **d**).

In *N. spumigena*, five classes of non-ribosomal peptides have been identified: linear spumigins [20,21], aeruginosins [7,22], pseudoaeruginosins [23], cyclic nodularins [24], and anabaenopeptins [16,20]. Spumigins (SPUs) are linear tetrapeptides (Figure 1a) with hydroxyphenyl lactic acid (Hpla^1^) in *N*-terminal position, Arg^4^ or Arg mimetics in *C*-terminus and (2*S*, 4*S*)-4-methylproline (MePro) or Pro in position 3. In the least conserved position 2, hydrophobic d-amino acid: homo-tyrosin (Htyr), homo-phenylalanine (Hph), Leu or Tyr were present. Aeruginosins (AERs) produced by *N. spumigena* have a unique feature of having short fatty acid chains in position 1 (Figure 1b). All of these peptides possess Tyr and a 2-carboxy-6-hydroxyoctahydroindole (Choi) in positions 2 and 3, respectively. Like in spumigins, in the *C*-terminus of aeruginosins, different Arg mimetics were found. The two known pseudoaeruginosins (NS1 and NS2) contain hexanoic acid^1^, Tyr^2^, MePro^3^, and argininal^4^ or argininol^4^ [23] (Figure 1c). Thus, they differ from aeruginosins in the residue, which occupies position 3. Nodularin, the hepatotoxic pentapeptide, is the most commonly studied *N. spumigena* metabolite; several variants of the compound with different degrees of methylation have been identified [24]. Anabaenopeptins (APs) are composed of a five-unit ring structure and the exocyclic amino acid linked with d-Lys through the ureido bound (Figure 1d). In many APs, a homo-amino acid is present in position 4 and an *N*-methylated amino acid in position 5. Anabaenopeptins produced by *N. spumigena* are characterized by the presence of Met or Ser in position 6 and are called nodulapeptins [20]. So far, the production of at least 87 APs by cyanobacteria has been documented, of these 22 were detected in *N. spumigena* [25].

In freshwater ecosystems, NRPs have been used to study the metabolic diversity (i.e., chemotypes) of potentially toxic cyanobacteria such as *Microcystis* [26,27] and *Planktothrix* [28,29]. There is evidence that within the same bloom event, conspecific strains producing different classes of NRPs and/or strains producing different structural variants of the same class of peptide co-exist [26,27]. As the profiles of the peptides are stable chemical markers, they have been used to distinguish phenotypically different sub-populations and trace their dynamics in freshwater ecosystems [28,30]. Recently, NRP profiles of brackish *N. spumigena* from the Baltic Sea (9 strains) and Australian (8 strains) waters were reported [7]. The authors concluded that the diversity of NRPs in the Baltic Sea was higher than in Australian waters. The Baltic Sea is one of the largest brackish water bodies in the world and is characterized by a strong horizontal salinity gradient. Blooms of *N. spumigena* occur along a salinity range of 5–15 PSU, however the geographical diversity of populations/sub-populations is unknown. 

Studies focusing on the diversity, distribution and dynamics of *N. spumigena* chemotypes (i.e., clusters of strains with similar NRP profile) will provide relevant insights into the ecology of summer cyanobacterial blooms in the Baltic Sea. In this study, we explored the different categories of chemotypes among 25 *N. spumigena* strains isolated from different locations in the Baltic Sea. The chemical diversity of the strains was determined based on the profile of 5 classes of NRPs belonging to spumigins, aeruginosins, pseudoaeruginosins, nodularins, and anabaenopeptins. The genetic diversity was assessed based on the sequences of the phycocyanin intergenic spacer and its flanking regions (*cpcBA-IGS*). The *cpc*BA-IGS sequences are characterized by a higher variability than the conserved 16S rRNA genes, and are thus suitable markers to explore the intraspecific variability in *N. spumigena* [31]. Finally, the grouping of the strains according to both chemical and genetic diversity was compared.

## 2. Results and Discussion

In the brackish Baltic Sea, cyanobacteria summer blooms are an abundant source of bioactive cyanobacterial peptides [25]. In the toxic *N. spumigena*, as in other cyanobacteria, the profiles of non-ribosomal peptides are strain specific [7,25]. Our study showed for the first time that chemical diversity of NRPs matches the genetic diversity of *N. spumigena*, regardless of geographical origin of the strains. We show that two core chemotypes/genetic clusters of this species are present in the Baltic Sea. 

### 2.1. Non-Ribosomal Peptides and Peptide Profiles of Baltic Nodularia spumigena

Qualitative variation of NRPs among the 25 *N. spumigena* strains (Table S1) was tremendous as 60 NRPs were detected (Table S2). These include 14 spumigins, 5 aeruginosins, 2 pseudoaeruginosins, 2 nodularins, and 36 anabaenopeptins (Table S2). Many of these peptides have been previously reported [7,16,20,21,22,23,25]. However, new proposed structures of 7 anabaenopeptins (APs) and one cyanopeptolin-like peptide were characterized in this study (Table S2 and Figures S1–S8). The number of peptides detected in each *N. spumigena* strain varied from 10 (CCNP1430, CCNP1426 and KAC13) to 35 (AV1) (Table S3). Spumigins (SPUs), nodularins (NPs) and APs were detected in all 25 *N. spumigena* strains, whereas aeruginosins were not found in seven of them (Figure S9 and Table S3). These findings agree with previous studies [21,22,23]. Production of pseudoaeruginosins NS1 (*m*/*z* 531) and NS2 (*m*/*z* 533) was confirmed only in six strains (CCNP1402, BY1, CCY9414, AV1, KAC71, and KAC87), indicating that in *N. spumigena* this class of peptides belongs to the most rare ones. Despite a scarce production of aeruginosins and pseudaeruginosins in *N. spumigena* [22,23], the presence of genes involved in the biosynthesis of aeruginosins has been documented in all strains examined from the Baltic strains including BY1, AV1 and CCY9414 [22,23]. The analysis of the *N. spumigena* CCY9414 genome additionally indicated the presence of cryptic gene clusters encoding other, yet unknown peptides to be discovered [32]. 

The number of SPUs in each *N. spumigena* strain varied from 4 to 11 (Figure S9 and Table S3). Of these, SPU E (*m*/*z* 611), SPU F (*m*/*z* 597) and SPU D *(m*/*z* 599) were most common. The number of SPUs with Pro in the third position was higher (9/14) than with MePro (5/14). In the *C*-terminal position, argininal (Argal), the reduced form of Arg, occurred more frequently (8/14) than other forms. These results confirm the frequency of Argal in the structure of SPUs for the strains AV1 and CCY9414 reported by Fewer et al. [21]. According to these authors, the synthesis of SPUs is catalyzed by two multidomain NRPS proteins, composed of one SpuA module and three SpuB modules. The last module of SpuB contains *C*-terminal reductase domain (R), which catalyses the release of spumigins as peptide aldehyde [21]. It was suggested that Arg-containing SPU B (*m*/*z* 627) might be an artifact formed during extraction procedure [21]. As LC-MS/MS analyses of this particular peptide in our study were not reproducible, we decided to exclude the peptide from our diversity study.

Aeruginosins produced by *N. spumigena* are characterized by the presence of short fatty acids in the *N*-terminus and l-Tyr in the second position [22]. Our study revealed the presence of only five AER variants (Table S2). In the *N*-terminus, they contain butanoic (*m*/*z* 559), hexanoic (*m*/*z* 603, 589, 587) or octanoic acid (*m*/*z* 615), but not acetic acid nor decanoic acid as identified in other *N. spumigena* strains [22]. In aeruginosins from other cyanobacteria, this position is occupied by Hpla or Pla [33]. In two *N. spumigena* strains, AV1 and CH307, Fewer et al. [22] identified 11 aeruginosin variants and depending on the presence of Argal or Argol residue, marked them as NAL or NOL, respectively. In four of the structures, pentose was located at Choi. Similarly to SPUs, in the structure of the identified AERs, in the C terminus, Argal was most common and occurred in three peptides, including the most abundant NAL2 (*m*/*z* 587). The preference of Argal in aeruginosin structure results from the presence of reductase domain in the *C*-terminal AerM module of the aeruginosin NRPS [22]. 

Pseudoaeruginosins, the third group of linear peptides produced by *N. spumigena*, were previously detected in 33 of the 62 examined strains from the Baltic Sea [23]. According to Liu et al. [23], NS1 and NS2 variants contained MePro in the third position and always occurred in pair. These observations were also made in our study, but the two compounds were detected in few strains (6/25), and only in those that produce APs with Ile in the exocyclic position (Figure S9 and Table S3), five from chemotype cluster A, CT_A, and one from chemotype cluster B sub-group B1, CT_B1). Based on the structure and the abundance of individual linear peptides, Liu et al. [23] divided *N. spumigena* strains into two groups: those producing SPUs with almost exclusively Pro in the third position and those producing mainly SPUs with MePro residue. In the latter strains, the presence of pseudoaeruginosins was revealed. Relative amounts of SPU with Pro and SPU with MePro in the Baltic *N. spumigena* strains were also roughly estimated in our studies (Table S4). In all pseudoaeruginosin-containing isolates, SPUs with MePro dominated or were at least as abundant as Pro-containing SPUs. However, the dominance of MePro in SPU was not always accompanied by production of pseudoaeruginosins (e.g., KAC7 or CCNP1427). On the other hand, in *N. spumigena* that produced solely or mainly Pro-containing SPUs, pseudoaeruginosins were not found (e.g., KAC64 or CCNP1401). Liu et al. [23] found a significant correlation between the presence of pseudoaeruginosins and the sum amount of MePro-containing spumigins and aeruginosins, and suggested that a hybrid structure of pseudoaeruginosins could result from the interactions between spumigin and aeruginosin biosynthetic pathways.

The structural variety of anabaenopeptins detected in Baltic *N. spumigena* is high (36, including seven new variants) (Table 1 and Table S2). This is in contrast to Australian strains in which only three APs were detected [7]. In this study, the majority (32) of APs produced by *N. spumigena* strains are nodulapeptins (NPs); one of the following residues was found in position 6: l-methionine or its oxidized forms (methionine sufloxide (Met(O)), methionine sulfone (Met(O_2_)), l-serine or O-acetylserine (Ser(Ac)). In APs from a summer bloom sample dominated by *N. spumigena* [25], and in 4 strains from cluster subgroup CT_B2, position 6 was occupied by Phe (Table 1). In position 3, we frequently observed Val (16) or Ile (7), but Met or its oxidized form (12) were also present. Position 4 of the Baltic *N. spumigena* was relatively conserved, and in all APs was occupied either by Hph (20) or by Hty (6). In position 5, MeHty (20), MeHph (12) or MeAla (4) were identified. All APs produced by Baltic *N. spumigena* have consistently only one of two possible kinds of residue in the exocyclic position: Ile or Phe [7]. Using *N. spumigena* strain CCY9414, Rouhiainen et al. [16] demonstrated that conserved residue in position 1 is determined by a single starter module in the NRPS enzyme complex and by substrate specificity of adenylation domain that catalyzes the activation of the first amino acid in the peptide. Our findings take this evidence a step further: In our study, AP variants with Ile in the side chain (position 1) dominated and were detected in 20 of the 25 strains, indicating a conserved chemical trait among *N. spumigena* Baltic populations (Table 1 and Table S3).

**Figure 2:**
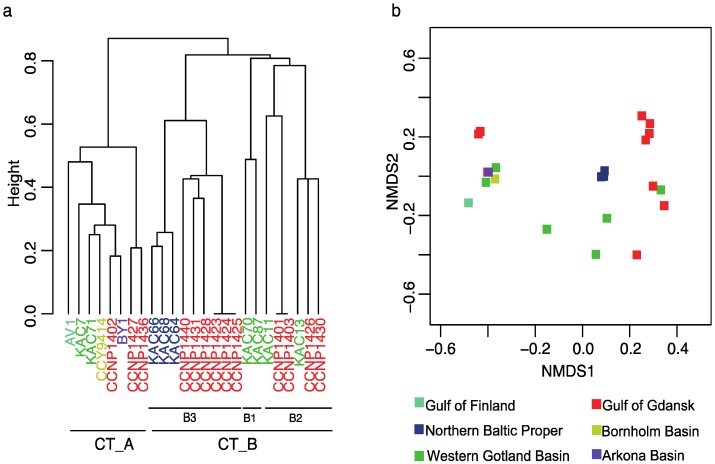
Cluster analyses (**a**) and ordination plot of non-metric multidimensional scaling (**b**, NMDS) based on Jaccard dissimilarity matrix of peptide profiles detected in *Nodularia spumigena* from the Baltic Sea. Two chemotype clusters were identified CT_A and CT_B. In CT_B, different sub-groups were found CT_B1, CT_B2 and CT_B3. Colors denote strains from Gulf of Finland (turquoise), Northern Baltic Proper (Askö, dark blue), Western Gotland Basin (green), Gulf of Gdansk (red), Bornholm Sea (yellow), and Arkona Sea (purple).

Based on the NRP profile, the Baltic *N. spumigena* strains were grouped into two core chemotype clusters CT_A and CT_B, with no apparent influence of the geographical origin of the strains (Figure 2, Figure S9 and Table S3). CT_A is the smallest group and consists of eight strains with the highest number of peptides (22–35), the highest frequency of pseudoaeruginosins, and where all APs are nodulapeptins (NPs). All NPs possess Ile in the exocyclic position and Hph in position 4 (Table 1). Therefore, including the conserved Lys in position 2, they all have three residues in common. High number of NP variants produced by strains from CT_A results from a variety of units in positions 4 and 6, which is probably related to a relaxed substrate specificity of adenylation domains involved in substitution of the units. In CT_A, strain AV1 produces the highest number of NPs and is the only strain where a new variant of cyanopeptolin-like peptide (*m*/*z* 848) was detected (Table S3). The fragmentation spectrum of the peptide contains some ion peaks indicating the presence of a sequence Apha + Phe + MeTyr characteristic for cyanopeptolins (Figure S8). This class of NRPs was identified in many freshwater cyanobacteria, mainly in *Microcystis* and *Planktothrix* [28,34], but we report the presence of this peptide in brackish *N. spumigena* for the first time.

Chemotype cluster CT_B was more heterogenous than CT_A and contained strains with a higher chemical diversity (i.e., many strains with unique features), but with a lower number of peptides (from 10 to 22) than strains from cluster A (Figure S9 and Table S3). CT_B included strains producing APs with Ile or Phe in exocyclic position and strains with or without aeruginosins. Within CT_B, three sub-groups, CT_B1–B3, differing in some features could be distinguished. Two strains that formed sub-group B1, KAC70 and KAC87 produced APs with Ile in the exocyclic position and all contained the same sequence of residues Ile^1^ + CO + Lys^2^ + Val^3^ + Hph^4^. KAC87 was the only strain from cluster B in which pseudoaeruginosin was found. In position 1 of APs produced by strains from CT_B2, both Ile and Phe could be found. However, all of these peptides possess Lys^2^, Hty^4^, MeAla^5^ and Phe^6^ in their structures, thus they shared the same four residues. As APs produced by representatives from CT_B2 did not contain Met or Ser/AcSer, they were not classified to nodulapeptins. In SPUs from CT_B2, modification in *N*-terminal Hpla residue (Hpla + 42), and with Leu (*m*/*z* 575) and Tyr (*m*/*z* 583) in position 2 were detected. APs produced by nine *N. spumigena* strains from CT_B3 were all characterized by the same three-amino acid sequence Phe^1^ + CO + Lys^2^ + Val^3^ (Table S2).

All classes of peptides included in this study are active against several key metabolic enzymes and their pharmaceutical potential has been frequently explored [23,33,35]. As was shown, several NRP variants are produced by *N. spumigena* individual strains and significant strain-specific differences in peptides profiles exist. Therefore, the utility of *N. spumigena* in biotechnology might be strain-dependent. The chemotype clustering approach in this study may facilitate the screening of potentially interesting strains with unique features. On the other hand, the chemical diversity of the strains can also have significant ecological implications, likely with a broad spectrum of functionality from the small (molecular, cellular) to the higher plankton community level with regard to antimicrobial and anti-grazing activity, competition.

### 2.2. PC-IGS Sequences and Phylogenetic Analyses of Baltic Nodularia spumigena 

**Figure 3:**
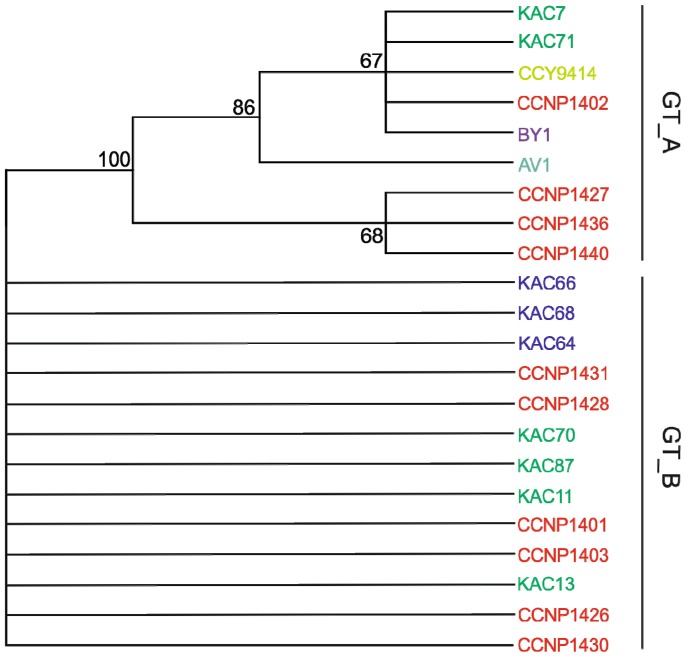
Maximum parsimony (MP) phylogenetic tree based on representative sequences of the *cpcBA-IGS* (587 bp). Bootstrap values were calculated (1000 replicated trees) and are displayed when greater than 0.5. Strains are divided in two genetic clusters, GT_A and GT_B. The strains CCNP1423, CCNP1424, CCNP1425 are not included in the tree as they were lost prior to genetic analyses. Colors denote strains from Gulf of Finland (turquoise), Northern Baltic Proper (Askö, dark blue), Western Gotland Basin (green), Gulf of Gdansk (red), Bornholm Sea (yellow), and Arkona Sea (purple).

Considering the 22 *N. spumigena* strains, phylogenetic analyses performed by neighbour-joining (NJ), maximum likelihood (ML) and maximum parsymony (MP) produced similar branching patterns (Figure 3 and Figure S10). We chose to present the MP phylogenetic tree as this method is recommended in phylogenetic reconstruction when analyzing highly similar sequences [36]. Two main genetic clusters GT_A and GT_B were identified in Baltic *N. spumigena* strains (Figure 3), with no apparent influence of geographical origin. Genetic cluster GT_A was heterogeneous with nine strains, including the extensively studied strains CCY9414, BY1, AV1, and KAC66 (see references therein). GT_B was homogeneous with thirteen strains. To show the phylogenetic congruence between previously published results and our results, we retrieved eight Baltic *N. spumigena* PC-IGS sequences deposited in GenBank. The obtained maximum likelihood tree on the phycocyanin operon sequences of the strains confirmed the separation of the Baltic *N. spumigena* into two clusters (Figure S11). Separation of planktonic *Nodularia* strains into two or more clusters on the basis of genotypic grouping using different genes (PC-IGS, rDNA-ITS, *het*R, *rpo*B, *rbc*LX) was previously reported with no consistency [8,11,12,37,38,39]. In view of these discrepancies, it was concluded that to determine the genetic diversity of cyanobacterial population, multiple gene loci and/or a higher number of isolates should be used. Our analysis includes similar strains (5) as in Janson and Granéli [38] showing similar genetic clustering for GT_B and their largest homogeneous (100% similarity) cluster.

### 2.3. N. spumigena Chemotypes vs. Genotypes

Few studies have tested genotype-phenotype relationships in cyanobacteria [13,40,41]. In these studies, phenotypic features include morphology or production of a specific group of secondary metabolites. For instance, the analysis of NRP profile in Baltic and Australian *N. spumigena* strains showed clear differences [7] that can potentially be explained by geographical distance or spatial isolation originating in unique peptide structures and NRP profiles [29]. It has been suggested that the significant difference in structural diversity of peptides among closely related strains can result from frequent recombination events and point mutations [17,42]. These evolutionary processes can lead to formation of new strains/subpopulations with their specific NRP profiles. In addition, the survival and dynamics of these subpopulations will strongly depend on biotic and abiotic selection forces, such as allelopathic interactions, grazing or infection by pathogens, nutrient regime, and light [28,40,41,43]. However, the reasons behind the high number of NRP variants detected in the Baltic *N. spumigena* remain to be elucidated. 

In this study, we compared the grouping of the Baltic *N. spumigena* strains based on chemical (NRPs) and genetic (PC-IGS) approaches (Figure 2 and Figure 3). As both chemical and genetic analyses grouped similarly *N. spumigena* strains from the Baltic Sea (with one exception of strain CCNP1440), we could define two main subpopulations inhabiting this sea. Some strains, isolated from the same location and year, belonged to different subpopulations. For instance, strains KAC7 (GT_A, CT_A) and KAC11 (GT_B, CT_B2), were isolated from Western Gotland basin in the same year (2000); and strains CCNP1427 (GT_A, CT_A) and CCNP1431 (GT_B, CT_B3) from the Gulf of Gdańsk in year 2012. In addition, other strains from various geographical locations of the Baltic Sea and isolated in different years (BY1 from Arkona Basin (1986); KAC7 from West Gotland Basin (2000); CCNP1402 from the Gulf of Gdańsk (2005)) belonged to the same *N. spumigena* subpopulation. Overall, these results of chemical and genetic diversity of *N. spumigena* in the Baltic Sea indicate that the blooms are not clonal, and the same subpopulations re-occurred in different years and parts of the Baltic Sea. The occurrence patterns of different subpopulations based on both chemotypes and genotypes have also been recorded for *Planktothrix* in a freshwater Lake Steinsfjorden in Norway for over 33 years [28]. Interestingly, changes in the dynamics of subpopulations were in response to environmental factors, suggesting potential distinct ecotypes. Noticeably, our recent study showed two *N. spumigena* strains classified here as two separate sub-groups, KAC11 (CT_B2) and KAC66 (CT_B3), which have different adaptation strategies to salinity changes [14].

*N. spumigena* is extensively distributed worldwide, from the northern to southern hemispheres, ranging from marine to freshwater bodies in both tropical and polar regions. The potential of using our CT/GT approach in other geographical regions would be of great relevance to characterize and determine the geographic range and frequency of these two subpopulations of *N. spumigena*. For instance, strain BY1 and CCNP1402 from cluster CT_A grouped together with the strains from Lake Iznik in Turkey [7]. This study is a major step in using chemical profiling to explore the conspecific diversity in *N. spumigena* with a higher resolution than with a sole genetic approach. The detailed phylogenetic analysis of similar *N. spumigena* chemotypes isolated from distant water bodies in the Baltic Sea expands the knowledge of the species history.

## 3. Materials and Methods 

### 3.1. Cultivation of Nodularia spumigena Strains

In total, 25 *N. spumigena* strains isolated from different parts of the Baltic Sea were analyzed (Table S1). Cyanobacteria were grown in a Z8 medium [44] supplemented with NaCl to salinity 7. The cultures were incubated at temperature 20 ± 2 °C and irradiance of 30 μmol photons m^−2^·s^−1^ with a light:dark cycle of 16:8 h. After one month of growth, filaments were harvested by filtration of 30 mL culture onto Whatman GF/C filters (Ø 2.5 cm). The samples were frozen and stored at −20 °C. To confirm the pattern of peptides produced by each *N. spumigena* strain, the culture experiments were replicated three times to obtain at least three independent extracts to be analyzed.

### 3.2. Extraction and LC-MS/MS Analysis of Peptides

The material collected on filters was extracted with 1 mL of 75% methanol in MilliQ water by 15 min bath sonication (Sonorex, Bandelin, Berlin, Germany). The samples were centrifuged (10 min, 10,000× *g*) and the supernatants were diluted with 6 mL of MilliQ water (to methanol content less than 20%). Then, the solid phase extraction was applied using 0.5 g Sep-Pak Vac C18 cartridges (Waters, Milford, MA, USA). The fraction eluted with 90% methanol was evaporated to dryness and re-dissolved in 0.5 mL of 50% methanol. After centrifugation, the supernatant was transferred to chromatographic vial and analyzed by LC-MS/MS.

LC-MS/MS analyses were performed on an Agilent 1200 (Agilent Technologies, Waldboronn, Germany) coupled to a triple-quadrupole mass spectrometer (5500 QTRAP, AB Sciex, Concord, ON, Canada). Extracts were separated on a Zorbax Eclipse XDB-C18 column (4.6 mm × 150 mm; 5 μm) (Agilent Technologies, Santa Clara, CA, USA) with a mobile phase composed of 5% acetonitrile in MilliQ water (A) and acetonitrile (B), both containing 0.1% formic acid. The flow rate was 0.6 mL·min^−1^, and the injection volume was 5 μL. The column temperature was 35 °C. The gradient elution started with 15% of mobile phase B, rising to 50% B over 10 min and then to 99% B in 5 min, held for 10 min, then decreased to 15% B in 2 min and held for 10 min to equilibrate the system.

Mass spectrometer operated in positive mode, with turbo ion spray (550 °C) voltage 5.5 kV and declustering potential of 80 V. Two types of MS/MS experiments were performed. In the first step, the information dependent acquisition method (IDA) was used and fragmentation spectra of all ions with *m*/*z* in a range 500–1000 and signal above the threshold of 500,000 cps were collected. The rough estimation of the relative amounts of peptides in extract was performed based on the intensity of the signal in extracted ion chromatogram. For selected ions, enhanced ion product mode (EIP) was used at a collision energy (CE 45–70 V) optimized to get the richest ion fragmentation spectrum. Data acquisition and processing were accomplished using Analyst QS^®^ 1.5.1 software.

To explore the diversity of non-ribosomal peptide profiles in 25 strains of Baltic *N. spumigena*, a dendogram based on hierarchical clustering and a non-metric multi-dimensional scaling (nMDS) was performed with a calculated Jaccard dissimilarity matrix (package Vegan) [45]. As input we used the strains with their respective presence or absence of the non-ribosomal peptides (*n* = 60). In the resulting dendogram and nMDS plot, strains with similar peptide composition are closely positioned. How well the analysis fit is indicated by the stress value (stress lower than 0.2 corresponds to a good representation). 

### 3.3. DNA Extraction and PC-IGS Sequencing

*N. spumigena* cells were harvested (~400 mg wet weight per sample) by centrifugation at 1600× *g* for 10 min, the supernatant was discarded and the pellet was frozen at −80 °C prior to extraction. DNA was extracted using the FastDNA^®^ SPIN Kit for Soil (MP Biomedicals, Santa Ana, CA, USA). The quality and quantity of extracted DNA was measured with SpectraMax^®^ i3 Platform (Molecular Devices LLC, Sunnyvale, CA, USA) equipped with SpectraDrop Micro-Volume Microplate (Molecular Devices, Sunnyvale, CA, USA). The PC-IGS region (*cpc*BA-IGS) was amplified using the primers PCβF and PCαR designed specifically for cyanobacteria [31]. The reactions (25 μL) contained 100 ng of DNA template, 5 pmol of each oligonucleotide primer and 12.5 μL of MyTaq™ Red Mix (Bioline Reagents Ltd., London, UK). Reaction mixtures were subjected to PCR conditions as described in [31]. PCR products were purified with ExtractMe DNA clean-up kit (Blirt S.A., Gdańsk, Poland) and sequenced (Genomed S.A., Warszawa, Poland) using forward primer. Sequences were deposited in GenBank under the accession numbers listed in Table S1.

The PC-IGS sequences (587 bp) of *N. spumigena* were aligned using MEGA version 6 [46] and the alignment was corrected manually. Neighbour-joining (NJ), maximum likelihood (ML) and maximum parsymony (MP) phylogenetic trees were constructed with MEGA version 6. For each tree, bootstrap analysis of 1000 replications was performed. For the ML phylogenetic tree analyses, the Kimura 2-parameter was found to be the best model fitting evolutionary substitution. MP was used with subtree pruning regrafting search algorithm and random tree addition. 

## 4. Conclusions

The analysis of five classes of non-ribosomal peptides: spumigins, aeruginosins, pseudoaeruginosins, nodularins, and anabaenopeptins, showed high chemical diversity in the Baltic *N. spumigena* populations. The identified chemotypes differ in the number and structure of the produced peptide variants. Only spumigins and nodularins were detected in all strains. Pseudoaeruginosins were found only in those six (of the 25) strains, which simultaneously produced spumigin variants with mainly MePro in position 3. These results support the hypothesis that hybrid structure of pseudoaeruginosins is a consequence of the interactions between spumigin and aeruginosin biosynthetic pathways [23]. In the structure of the 36 anabaenopeptins identified in the Baltic *N. spumigena* strains, apart from the conserved Lys, the variety of amino acids in position 1 and 4 was limited to two residues. The high number of detected variants was mainly due to a variety of unites in position 6, which indicates the lowest substrate specificity of the adenylation domain catalyzing substitution of amino acid in this position. Based on the NRP profiles reported for strains isolated over 30-years and from different geographical locations in the Baltic Sea, we could detect the co-existence of two main *N. spumigena* chemotypes. The analysis of PC-IGS sequences gave similar grouping of the strains, suggesting a strong link between chemotype and genotype. Our study established the use of chemical profiling to find intraspecific variability in *N. spumigena* subpopulations, contributing to the characterization of subpopulations not solely at the genetic level but also at the chemical level. Thus, future chemotypic and genetic screening of a higher number *N. spumigena* strains from different geographical regions should add new insight into the history and diversity of *N. spumigena* in aquatic ecosystems in the world.

## Figures and Tables

**Table 1:** Anabaenopeptins (APs) produced by *Nodularia spumigena* strains from the Baltic Sea. APs are likely to structure chemotype clustering. Two chemotype clusters were identified CT_A and CT_B. In CT_B, different sub-groups were found CT_B1, CT_B2 and CT_B3. New peptide structures are denoted with an asterisk.

Chemotype Cluster	*N. spumigena* Strain	Peptide *m*/*z*	Amino Acids Components					
			1	2	3	4	5	6
CT_A	CCNP1402, BY1, CCY9414, AV1, CCNP1427, CCNP1436, KAC7, KAC71	932	Ile	Lys	MetO	Hph	MeHty	MetO
		916a	Ile	Lys	MetO	Hph	MeHty	Met
		914	Ile	Lys	MetO	Hph	MeHty	AcSer
		900b	Ile	Lys	Met	Hph	MeHty	Met
		898a	Ile	Lys	Met	Hph	MeHty	AcSer
		898b	Ile	Lys	MetO	Hph	MeHph	AcSer
		884a	Ile	Lys	Met	Hph	MeHph	Met
		882a	Ile	Lys	Met	Hph	MeHph	AcSer
		882b	Ile	Lys	Ile	Hph	MeHty	Met
		880	Ile	Lys	Ile	Hph	MeHty	AcSer
		872	Ile	Lys	MetO	Hph	MeHty	Ser
		856a	Ile	Lys	Met	Hph	MeHty	Ser
		*840	Ile	Lys	Met	Hph	MeHph	Ser
	CCNP1402, CCY9414, AV1, KAC71	930	Ile	Lys	MetO_2_	Hph	MeHty	AcSer
	CCNP1402, BY1, CCY9414, CCNP1427, CCNP1436, KAC7, KAC71	856b	Ile	Lys	MetO	Hph	MeHph	Ser
	CCNP1402, BY1, AV1, CCNP1427, CCNP1436, KAC7, KAC71	*822	Ile	Lys	Ile	Hph	MeHph	Ser
	KAC71	824	Ile	Lys	Val	Hph	MeHty	Ser
	AV1	868	Ile	Lys	Val	Hph	MeHty	Met
		866	Ile	Lys	Val	Hph	MeHty	AcSer
		*864	Ile	Lys	Ile	Hph	MeHph	AcSer
		*850	Ile	Lys	Val	Hph	MeHph	AcSer
CT_B1	KAC70, KAC87	884c	Ile	Lys	Val	Hph	MeHty	MetO
		868	Ile	Lys	Val	Hph	MeHty	Met
		866	Ile	Lys	Val	Hph	MeHty	AcSer
		*852	Ile	Lys	Val	Hph	MeHph	Met
		824	Ile	Lys	Val	Hph	MeHty	Ser
		*808a	Ile	Lys	Val	Hph	MeHph	Ser
	KAC87	*850	Ile	Lys	Val	Hph	MeHph	AcSer
CT_B2	KAC 11	808b	Ile	Lys	Ile	Hty	MeAla	Phe
		794	Ile	Lys	Val	Hty	MeAla	Phe
	CCNP1401, CCNP1403, CCNP1426, CCNP1430, KAC13	842	Phe	Lys	Ile	Hty	MeAla	Phe
		828	Phe	Lys	Val	Hty	MeAla	Phe
CT_B3	CCNP1440, CCNP1431, CCNP1428, CCNP1423, CCNP1424, CCNP1425, KAC64, KAC66, KAC68	934	Phe	Lys	Val	Hty	MeHty	MetO
		918	Phe	Lys	Val	Hph	MeHty	MetO
		902	Phe	Lys	Val	Hph	MeHty	Met
		900a	Phe	Lys	Val	Hph	MeHty	AcSer
		884b	Phe	Lys	Val	Hph	MeHph	AcSer
	CCNP1423, CCNP1424, CCNP1425, CCNP1428, CCNP1431, CCNP1440, KAC64, KAC66	916b	Phe	Lys	Val	Hty	MeHty	AcSer
	CCNP1431, CCNP1440, KAC64, KAC66, KAC68	*886	Phe	Lys	Val	Hph	MeHph	Met
	KAC66, KAC68	858	Phe	Lys	Val	Hph	MeHTyr	Ser

## Supplementary Materials

The following are available online at www.mdpi.com/1660-3397/14/11/209/s1, Figures S1–S8: Fragmentation spectra of the new anabaenopeptin variants (7) and the cyanopeptolin-like peptide (1) detected in this study, Figure S9: Heatmap representing the presence/absence of non-ribosomal peptides (NRPs) in each *N. spumigena* strain from the Baltic Sea (*n* = 60). NRPs are abbreviated as spumigins (SPUs), aeruginosins (AERs), pseudoaeruginosins (NS), anabaenopeptins (APs) and cyanopeptolin-like peptide (CYP). Novel peptide structures are denoted with an asterisk, Figure S10: Neighbour-joining (NJ) (a) and Maximum-likelihood (ML) (b) phylogenetic trees based on representative sequences of the *cpcBA-IGS* (587 bp). Bootstrap values were calculated (1000 replicated trees) and are displayed when greater than 0.5. Strains are divided in two genetic clusters, GT_A and GT_B, Figure S11: Maximum-likelihood (ML) phylogenetic tree on the phycocyanin operon (PC-IGS) sequences (471 bp) of the *N. spumigena* strains in this study (marked in black) and references sequences retrieved from NCBI (marked in blue). Clades designated in this study are indicated (GT_A and GT_B). Phylogenetic relationships were bootstrapped 1000 times, Table S1: *Nodularia spumigena* strains isolated from different regions of the Baltic Sea and used for chemical and genetic analyses. DNA sequences used in alignment and phylogenetic analyses can be retrieved from GenBank. AV1 and BY1: Dept. of Applied Chemistry and Microbiology, University of Helsinki, Finland; KAC: Kalmar Algae Collection, Linnæus University, Sweden; CCNP: Culture Collection of Northern Poland, Dept. of Marine Biotechnology, Gdansk University; CCY: Culture Collection Yerseke, The Royal Netherlands Institute for Sea Research, The Netherlands, Table S2: Peptides detected in the Baltic *Nodularia spumigena* strains. New peptide structures are denoted with an asterisk, Table S3: Peptides produced by the Baltic *Nodularia spumigena* strains. Two chemotype clusters were identified CT_A and CT_B. In CT_B, different sub-groups were found CT_B1, CT_B2 and CT_B3. In the table, the peptides are characterized by *m*/*z* values of their molecular ions. New peptide structures are denoted with an asterisk, Table S4: Relative intensity of extracted ion peaks of spumigins produced by the Baltic *Nodularia spumigena* strains. The type of residue in position 3 (Pro/MePro) is indicated. Spumigins detected in trace amounts, at ion intensity lower than 10^5^ are marked with letter t.

## Abbreviations

The following abbreviations are used in this manuscript:

AcSer	acetylserine
Agm	agmatine
Bu	butanoic acid
Hex	hexanoic acid
Hpla	hydroxyphenyl lactic acid
Hph	homophenylalanine
Hty	homotyrosine
MePro	metylproline
MetO	methionine sufloxide
Met(O_2_)	methionine sulfone
MeHty	methylhomotyrosine
Oct	octanoic acid

## References

[1] Jones G.J., Blackburn S.I., Parker N.S. (1994). A toxic bloom of *Nodularia spumigena* Mertens in Orielton Lagoon, Tasmania. Aust. J. Mar. Freshw. Res..

[2] John J., Kemp A. (2006). Cyanobacterial blooms in the wetlands of the Perth region, taxonomy and distribution: An overview. J. R. Soc. West. Aust..

[3] McGregor G.B., Stewart I., Sendall B.C., Sadler R., Reardon K., Carter S., Wruck D., Wickramasinghe W. (2012). First report of a toxic *Nodularia spumigena* (Nostocales/Cyanobacteria) bloom in sub-tropical Australia. Phycological and public health investigations. Int. J. Environ. Res. Public Health.

[4] Sivonen K., Kononen K., Carmichael W., Dahlem A.M., Rinehart K.L., Kiviranta J., Niemela S.I. (1989). Occurrence of the hepatotoxic cyanobacterium *Nodularia spumigena* in the Baltic Sea and structure of the toxin. Appl. Environ. Microbiol..

[5] Karjalainen M., Engström-Öst J., Korpinen S., Peltonen H., Pääkkönen J.P., Rönkkönen S., Suikkanen S., Viitasalo M. (2007). Ecosystem consequences of cyanobacteria in the northern Baltic Sea. AMBIO.

[6] Sotton B., Domaizon I., Anneville O., Cattanéo F., Guillard J. (2015). Nodularin and cylindrospermopsin: A review of their effects on fish. Rev. Fish Biol. Fisher..

[7] Mazur-Marzec H., Kaczkowska M.J., Błaszczyk A., Akcaalan R., Spoof L., Meriluoto J. (2013). Diversity of peptides produced by *Nodularia spumigena* from various geographical regions. Mar. Drugs.

[8] Lehtimäki J., Lyra C., Suomalainen S., Sundman P., Rouhiainen L., Paulin L., Salkinoja-Salonen M., Sivonen K. (2000). Characterization of *Nodularia* strains, cyanobacteria from brackish waters, by genotypic and phenotypic methods. Int. J. Syst. Evol. Microbiol..

[9] Congestri R., Capucci E., Albertano P. (2003). Morphometric variability of the genus *Nodularia* (Cyanobacteria) in Baltic natural communities. Aquat. Microb. Ecol..

[10] Komárek J., Hübel M., Hübel H., Šmarda J. (1993). The *Nodularia* studies 2. Taxonomy. Algol. Stud..

[11] Laamanen M.J., Gugger M.F., Lehtimäki J., Haukka K., Sivonen K. (2001). Diversity of toxic and non-toxic *Nodularia* isolates (Cyanobacteria) and filaments from the Baltic Sea. Appl. Environ. Microbiol..

[12] Barker G.L., Handley B.A., Vacharapiyasophon P., Stevens J.R., Hayes P.K. (2000). Allele-specific PCR shows that genetic exchange occurs among genetically diverse *Nodularia* (Cyanobacteria) filaments in the Baltic Sea. Microbiology.

[13] Řeháková K., Mareš J., Lukkešová A., Zapomělová E., Bernardová K., Hrouzek P. (2014). *Nodularia* (Cyanobacteria, Nostocaceae): A phylogenetically uniform genus with variable phenotypes. Phytotaxa.

[14] Bertos-Fortis M., Klotz F., Mazur-Marzec H., Legrand C. (2016). Phenotypic plasticity in brackish water cyanobacteria: Survival at any cost under salinity changes. Evolution.

[15] Moffitt M.C., Neilan B.A. (2004). Characterization of the nodularin synthetase gene cluster and proposed theory of the evolution of cyanobacterial hepatotoxins. Appl. Environ. Microbiol..

[16] Rouhiainen L., Jokela J., Fewer D.P., Urmann M., Sivonen K. (2010). Two alternative starter modules for the non-ribosomal biosynthesis of specific anabaenopeptin variants in *Anabaena* (Cyanobacteria). Chem. Biol..

[17] Meyer S., Kehr J.K., Mainz A., Dehm D., Petras D., Süssmuth R.D., Dittmann E. (2016). Biochemical dissection of the natural diversification of microcystin provides lessons for synthetic biology of NRPS. Cell. Chem. Biol..

[18] Amoutzias G.D., Chaliotis A., Mossialos D. (2016). Discovery strategies of bioactive compounds synthesized by nonribosomal peptide synthetases and type-I polyketide synthases derived from marine microbiomes. Mar. Drugs.

[19] Briand E., Bormans M., Gugger M., Dorrestein P.C., Gerwick W.H. (2016). Changes in secondary metabolic profiles of *Microcystis aeruginosa* strains in response to intraspecific interactions. Environ. Microbiol..

[20] Fujii K., Sivonen K., Adachi K., Noguchi K., Shimizu Y., Sano H., Hirayama K., Suzuki M., Harada K.-I. (1997). Comparative study of toxic and non-toxic cyanobacterial products: Novel peptides from toxic *Nodularia spumigena* AV1. Tetrahedron Lett..

[21] Fewer D.P., Jokela J., Rouhiainen L., Wahlsten M., Koskenniemi K., Stal L., Sivonen K. (2009). The non-ribosomal assembly and frequent occurrence of the protease inhibitor spumigin in the bloom-forming cyanobacterium *Nodularia spumigena*. Mol. Microbiol..

[22] Fewer D.P., Jokela J., Paukku E., Österholm J., Wahlsten M., Permi P., Aitio O., Rouhiainen L., Gomeez-Saez G.V., Sivonen K. (2013). New structural variants of aeruginosins produced by the toxic bloom forming cyanobacterium *Nodularia spumigena*. PLoS ONE.

[23] Liu L., Budnjo A., Jokela J., Haug B.E., Fewer D.P., Wahlsten M., Rouhiainen L., Permi P., Fossen T., Sivonen K. (2014). Pseudoaeruginosis, non-ribosomal peptides in *Nodularia spumigena*. ACS Chem. Biol..

[24] Rinehart K.L., Harada K.I., Namikoshi M., Chen C., Harvis C.A., Munroe M.H.G., Blunt J.W., Mulligan P.E., Beasley V.R., Dahlem A.M. (1988). Nodularin, microcystin and the configuration of Adda. J. Am. Chem. Soc..

[25] Spoof L., Błaszczyk A., Meriluoto J., Cegłowska M., Mazur-Marzec H. (2016). Structures and activity of new anabaenopeptins produced by Baltic Sea cyanobacteria. Mar. Drugs.

[26] Welker M., Christiansen G., van Döhren H. (2004). Diversity of coexisting *Planktothrix* (Cyanobacteria) chemotypes deduced by mass spectral analysis of microcystins and other oligopeptides. Arch. Microbiol..

[27] Martins J., Saker M.L., Moreira C., Welker M., Fastner J., Vasconcelos V.M. (2009). Peptide diversity in strains of the cyanobacterium *Microcystis aeruginosa* isolated from Portuguese water supplies. Appl. Microbiol. Biotechnol..

[28] Rohrlack T., Skulberg R., Skulberg O.M. (2009). Distribution of oligopeptidechemotypes of the cyanobacterium *Planktothrix* and their persistence in selected lakes in Fennoscandia. J. Phycol..

[29] Kurmayer R., Blom J.F., Deng L., Pernthaler J. (2015). Integrating phylogeny, geographic niche partitioning and secondary metabolite synthesis in bloom-forming *Planktothrix*. ISME J..

[30] Agha R., Quesada A. (2014). Oligopeptides as biomarkers of cyanobacterial subpopulations. Toward an understanding of their biological role. Toxins.

[31] Neilan B.A., Jacobs D., Goodman A.E. (1995). Genetic Diversity and phylogeny of toxic cyanobacteria determined by DNA polymorphisms within the phycocyanin locus. Appl. Environ. Microbiol..

[32] Voß B., Bolhuis H., Fewer D.P., Kopf M., Möke F., Haas F., El-Shehawy R., Hayes P., Bergman B., Sivonen K. (2013). Insights into the physiology and ecology of the brackish-water-adapted cyanobacterium *Nodularia spumigena* CCY9414 based on a genome-transcriptome analysis. PLoS ONE.

[33] Ersmark K., Del Valle J.R., Hanessian S. (2008). Chemistry and Biology of the aeruginosin family of serine protease inhibitors. Angew. Chem. Int. Ed..

[34] Czarnecki O., Henning M., Lippert I., Welker M. (2006). Identyfication of peptide metabolites of *Microcystis* (Cyanobacteria) that inhibit trypsin-like activity in planktonic herbivorous *Daphnia* (Cladocera). Environ. Microbiol..

[35] Ishida K., Okita Y., Matsuda H., Murakami M. (1999). Aeruginosins, protease inhibitors from the cyanobacterium *Microcystis aeruginosa*. Tetrahedron.

[36] Mount D.W. (2004). Bioinformatics: Sequence and Genome Analysis.

[37] Bolch C.J.S., Orr P.T., Jones G.J., Blackburn S.I. (1999). Genetic, morphological and toxicological variation among globally distributed strains of *Nodularia* (Cyanobacteria). J. Phycol..

[38] Janson S., Granéli E. (2002). Phylogenetic analyses of nitrogen-fixing cyanobacteria from Baltic Sea reveal sequence anomalies in the phycocyanin operon. Int. J. Syst. Evol. Microbiol..

[39] Lyra C., Laamanen M., Lehtimäki J.M., Surakka A., Sivonen K. (2005). Benthic cyanobacteria of the genus *Nodularia* are non-toxic, without vacuoles, able to glide and genetically more diverse than planktonic *Nodularia*. Int. J. Syst. Evol. Microbiol..

[40] Rounge T.B., Rohrlack T., Decenciere B., Edvardsen B., Kristensen T., Jakobsen K.S. (2010). Subpopulation differentiation associated with nonribosomal peptide synthetase gene cluster dynamics in the cyanobacterium *Planktothrix* spp.. J. Phycol..

[41] Sogge H., Rohrlack T., Rounge T.B., Sønstebe J.H., Tooming-Klunderud A., Kristensen T., Jakobsen K.S. (2013). Gene flow, recombination, and selection in cyanobacteria: Population structure of geographically related *Planktothrix* freshwater strains. Appl. Environ. Microbiol..

[42] Tooming-Klunderud A., Fewer D.P., Rohrlack T., Jokela J., Rouhiainen L., Sivonen K., Kristensen T., Jakobsen K.J. (2008). Evidence for positive selection acting on microcystin synthetase adenylation domains in three cyanobacterial genera. BMC Evol. Biol..

[43] Suikkanen S., Fistarol G.O., Granéli E. (2004). Allelopathic effects of the Baltic cyanobacteria *Nodularia spumigena*, *Aphanizomenon flos-aquae* and *Anabaena lemmermannii* on algal monocultures. J. Exp. Mar. Biol. Ecol..

[44] Kotai J. (1972). Introduction for Preparation of Modified Nutrient Solution Z8 for Algae.

[45] CRAN—Package Vegan. http://CRAN.R-project.org/package=vegan.

[46] Tamura K., Stecher G., Peterson D., Filipski A., Kumar S. (2013). MEGA6: Molecular evolutionary genetics analysis version 6.0. Mol. Biol. Evol..

