# Supplementary material for: Chemical and Genetic Diversity of Nodularia spumigena from the Baltic Sea
Source: Mar Drugs. 2016 Nov 10;14(11):209. doi: 10.3390/md14110209 (PMC5128752; doi:10.3390/md14110209)
Supplement: Supplementary file 1 [file marinedrugs-14-00209-s001.doc]

Supplementary Material: Chemical and Genetic Diversity of *Nodularia spumigena* from the Baltic Sea

Hanna Mazur-Marzec, Mireia Bertos-Fortis, Anna Toruńska-Sitarz, Anna Fidor and
Catherine Legrand

**Figure S1**. Ion product mass spectra of anabaenopeptin AN885 Phe + CO + [Lys + Val + Hph + MeHph + Met] with [M + H] ion at *m*/*z* 886. The mass signals were assigned to the following fragments: 868 [M + H-H_2_O], 858 [M + H-CO], 840 [M + H-H_2_O-CO], 739 [M + H-Phe], 725 [M + H-Hph], 721 [M + H-Phe-H_2_O], 707 [M + H-Hph-H_2_O], 695 [M + H-(Phe + CO)], 608 [M + H-Phe-Met], 479 [M + H-Phe-(Hph + Val)], 387 [Hph + Val + Lys-H], 337 [MeHph + Hph + H], 307 [MeHph + Met + H], 148 MeHph, 120 Phe, 84 Lys.

**Figure S2**. Ion product mass spectra of anabaenopeptin AN863 Ile + CO + [Lys + Ile + Hph + MeHph + AcSer] with [M + H] ion at *m*/*z* 864. The mass signals were assigned to the following fragments: 846 [M + H_2_O], 836 [M + H-CO], 818 [M + H-CO-H_2_O], 804 [M + H-CH3COOH (from AcSer)], 786 [M + H-CH3COOH (from AcSer)-H_2_O], 776 [M + H-CH3COOH (from AcSer)-CO], 751 [M + H-Ile], 733 [M + H-Ile-H_2_O], 705 [M + H-Ile-H_2_O-CO], 707 [M + H-(Ile + CO)], 703 [M + H-Hph], 685 [M + H-Hph-H_2_O], 673 [M + H-Ile-H_2_O-CH3COOH (from AcSer)], 647 [M + H-(Ile + CO)-CH3COOH (from AcSer)], 643 [M + H-Hph-CH3COOH (from AcSer)], 629 [M + H-MeHph-CH3COOH (from AcSer)], 611 [M + H-MeHph-CH3COOH (from AcSer)-H_2_O], 477 [M + H-Ile-(Hph + Ile)], 450 [MeHph + Hph + Ile + H], 417 [M + H-Ile-(Hph + Ile)-CH3COOH (from AcSer)], 337 [MeHph + Hph + H], 305 [MeHph + AcSer + H], 148 MeHph, 84 Lys.

**Figure S3**. Ion product mass spectra of anabaenopeptin AN851 Ile + CO + [Lys + Val + Hph + MeHph + Met] with [M + H] ion at *m*/*z* 852. The mass signals were assigned to the following fragments: 834 [M + H-H_2_O], 824 [M + H-CO], 806 [M + H-H_2_O-CO], 753 [M + H-Val], 739 [M + H-Ile], 721 [M + H-Ile-H_2_O] or [M + H-Met], 703 [M + H-Met-H_2_O], 695 [M + H + (CO + Ile)], 691 [M + H-Hph], 677 [M + H-MeHph], 663 [M + H-Hph-CO], 564 [M + H-MeHph-Ile], 546 [M + H-MeHph-Ile-H_2_O], 479 [M + H-Ile-(Hph + Val)], 417 [Hph + Val + Lys + CO + H], 307 [MeHph + Hph + H], 307 [MeHph + Met + H], 148 MeHph, 84 Lys.

**Figure S4.** Ion product mass spectra of anabaenopeptin AN849 Ile + CO + [Lys + Val + Hph + MeHph + AcSer] with [M + H] ion at *m*/*z* 850. The mass signals were assigned to the following fragments: 832 [M + H-H_2_O], 822 [M + H-CO], 804 [M + H-H_2_O-CO], 790 [M + H-CH_3_COOH (from AcSer)], 772 [M + H-CH_3_COOH (from AcSer)-H_2_O], 762 [M + H-CH_3_COOH (from AcSer)-CO], 737 [M + H-Ile], 719 [M + H-Ile-H_2_O], 693 [M + H-(Ile + CO)], 689 [M + H-Hph], 633 [M + H-(Ile + CO)-CH_3_COOH (from AcSer)], 590 [M + H-Val-Hph], 544 [Hph + Val + Lys + CO + Ile-H], 477 [M + H-Ile-(Hph + Val)], 387 [Hph + Val + Lys + H], 337 [MeHph + Hph + H], 305 [MeHph + AcSer + H], 148 MeHph, 84 Lys.

**Figure S5.** Ion product mass spectra of anabaenopeptin AN839 Ile + CO + [Lys + Met + Hph + MeHph + Ser] with [M + H] ion at *m*/*z* 840. The mass signals were assigned to the following fragments: 822 [M + H-H_2_O], 812 [M + H-CO], 794 [M + H-H_2_O-CO], 727 [M + H-Ile], 709 [M + H-Ile-H_2_O] or [M + H-Met], 691 [M + H-Met-H_2_O], 683 [M + H-(Ile + CO)], 679 [M + H-Hph], 665 [M + H-MeHph], 647 [M + H-MeHph-H_2_O], 548 [M + H-(Hph + Met)], 530 [M + H-(Hph + Met)-H_2_O], 468 [MeHph + Hph + Met + H], 435 [M + H-Ile-(Hph + Met)], 337 [MeHph + Hph + H], 263 [MeHph + Ser + H], 148 MeHph, 84 Lys.

**Figure S6.** Ion product mass spectra of anabaenopeptin AN821 Ile + CO + [Lys + Ile + Hph + MeHph + Ser] with [M + H] ion at *m*/*z* 822. The mass signals were assigned to the following fragments: 804 [M + H-H_2_O], 794 [M + H-CO], 776 [M + H-H_2_O-CO], 709 [M + H-Ile], 691 [M + H-Ile-H_2_O], 665 [M + H-(CO + Ile)], 661 [M + H-Hph], 647 [M + H-(CO + Ile)-H_2_O] or 647 [M + H-MeHph], 643 [M + H-Hph-H_2_O], 548 [M + H-Ile-Hph], 530 [M + H-Ile-Hph-H_2_O], 450 [MeHph + Hph + Ile + H], 435 [M + H-Ile-(Hph + Ile)], 373 [Ser + Lys + CO + Ile + H], 337 [Hph + MeHph + H], 263 [MeHph + Ser + H], 148 MeHph, 84 Lys.

**Figure S7.** Ion product mass spectra of anabaenopeptin AN807 Ile + CO + [Lys + Val + Hph + MeHph + Ser] with [M + H] ion at *m*/*z* 808. The mass signals were assigned to the following fragments: 790 [M + H-H_2_O], 780 [M + H-CO], 762 [M + H-H_2_O-CO], 709 [M + H-Val], 695 [M + H-Ile], 677 [ + H + Ile-H_2_O], 651 [M + H-(Ile + CO)], 633 [M + H-MeHph], 615 [M + H-MeHph-H_2_O], 520 [M + H-Ile-MeHph], 435 [M + H-Ile-(Hph + Val)], 337 [MeHph + Hph + H], 263 [MeHph + Ser + H], 148 MeHph, 84 Lys.

**Figure S8**. Ion product mass spectra of cyanopeptolin-like peptide with [M + H] ion at *m*/*z* 848. The mass signals were assigned to the following fragments: 717 [M + H-Ile-H_2_O], 657 [M + H-MeHty], 434 [Ahp + Phe + MeHty + H-H_2_O], 243 [Ahp + Phe + H-H_2_O], 215 [Ahp + Phe + H-H_2_O-CO], 164 MeHtyr.


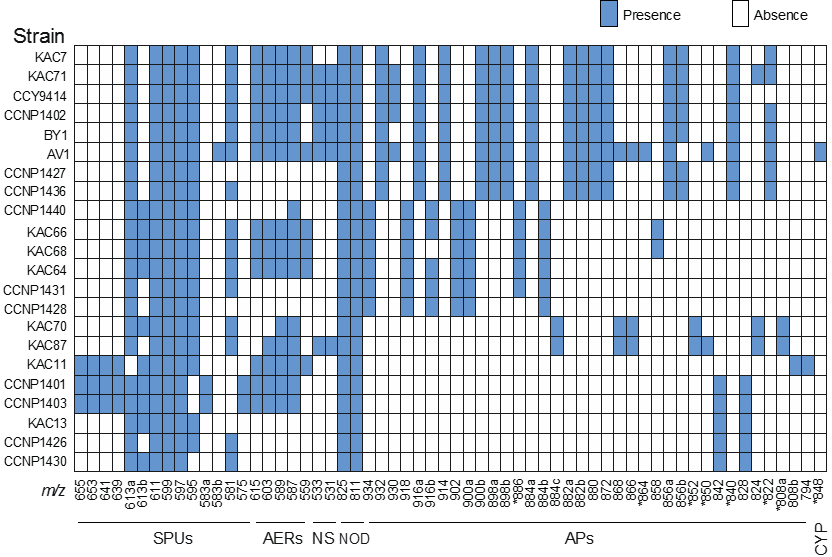


**Figure S9.** Heatmap representing the presence/absence of non-ribosomal peptides (NRPs) in each N. spumigena strain from the Baltic Sea (n=60). NRPs are abbreviated as spumigins (SPUs), aeruginosins (AERs), pseudoaeruginosins (NS), anabaenopeptins (APs) and cyanopeptolin-like peptide (CYP). New peptide structures are denoted with an asterisk.

(a)


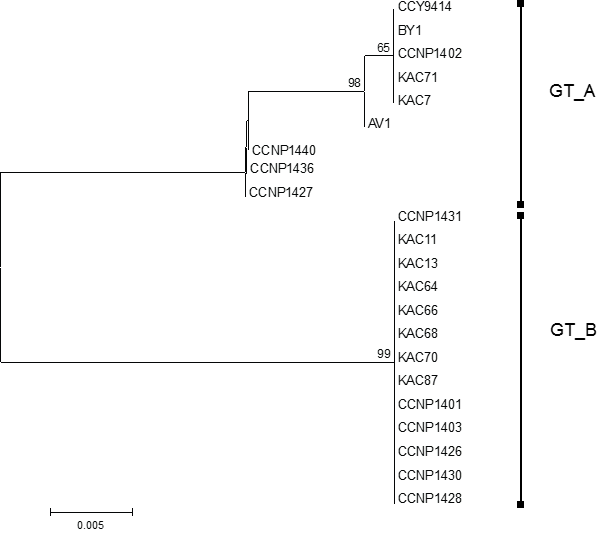


(**b**)


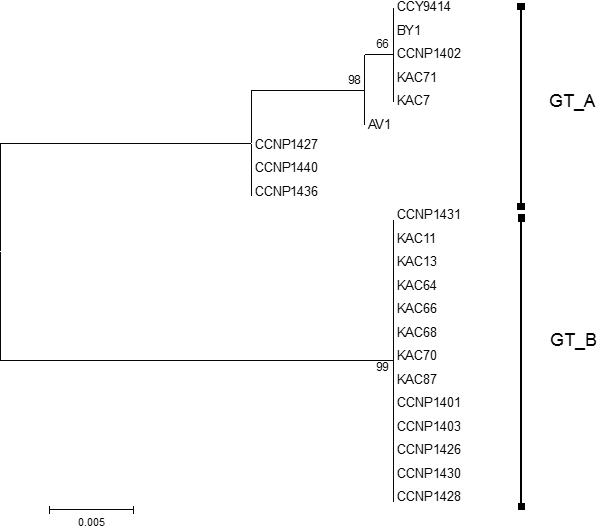


**Figure S10**. Neighbour-joining (NJ) (**a**) and Maximum-likelihood (ML) (**b**) phylogenetic trees based on representative sequences of the *cpcBA-*IGS (587 bp). Bootstrap values were calculated (1000 replicated trees) and are displayed when greater than 0.5. Strains are divided in two genetic clusters, GT_A and GT_B.


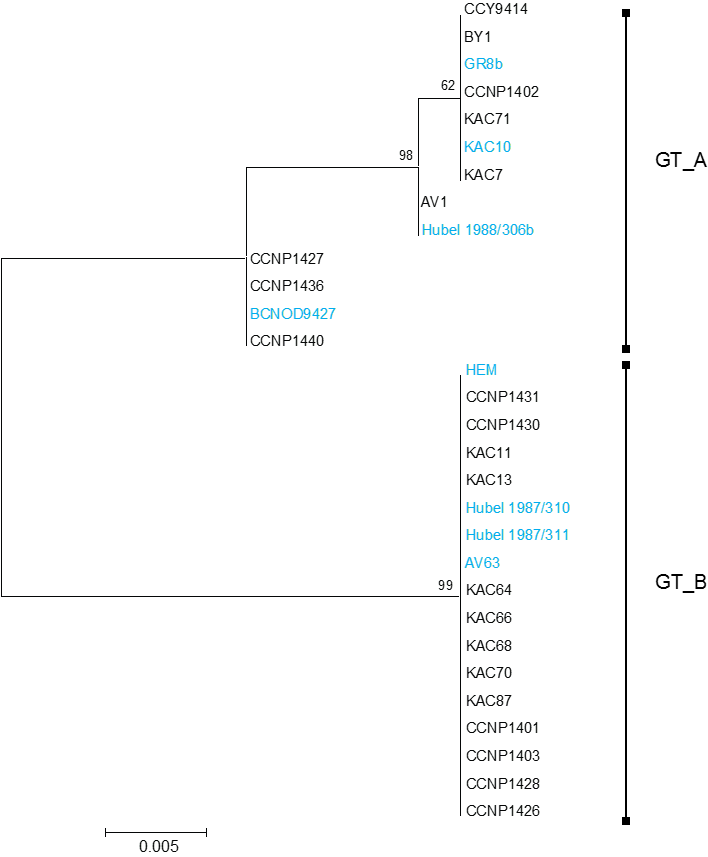


**Figure S11**. Maximum-likelihood (ML) phylogenetic tree on the phycocyanin operon (PC-IGS) sequences (471 bp) of the *N. spumigena* strains in this study (marked in black) and references sequences retrieved from NCBI (marked in blue). Clades designated in this study are indicated (GT_A and GT_B). Phylogenetic relationships were bootstrapped 1000 times.

**Table S1.** *Nodularia spumigena* strains isolated from different regions of the Baltic Sea and used for chemical and genetic analyses. DNA sequences used in alignment and phylogenetic analyses can be retrieved from GenBank. AV1 and BY1: Dept. of Applied Chemistry and Microbiology, University of Helsinki, Finland; KAC: Kalmar Algae Collection, Linnæus University, Sweden; CCNP: Culture Collection of Northern Poland, Dept. of Marine Biotechnology, Gdansk University; CCY: Culture Collection Yerseke, The Royal Netherlands Institute for Sea Research, The Netherlands.

| ***N. spumigena* Strain Code** | **Area of Isolation (Baltic Sea)** | **Lat. Lon.** | **Isolation Year** | **GenBank**  **Accesssion Number** |
| --- | --- | --- | --- | --- |
| AV1 | Gulf of Finland |  | 1987 | KX889414 |
| KAC64 | Northern Baltic Proper | 58°49.5′ N, 17°39′ E | 1996 | KX889399 |
| KAC66 |  | 58°49.5′ N, 17°39′ E | 1996 | KX889400 |
| KAC68 |  | 58°49.5′ N, 17°39′ E | 1996 | KX889401 |
| KAC7 | Western Gotland Basin |  | 2000 | KX889396 |
| KAC11 |  |  | 2000 | KX889397 |
| KAC13 |  |  | 1997 | KX889398 |
| KAC70 |  |  | 2000 | KX889402 |
| KAC71 |  | 57°1.6′ N,16°30.35′ E | 2001 | KX889403 |
| KAC87 |  | 56°55.85′ N, 17°03.64′ E | 2011 | KX889404 |
| CCNP1401 | Gulf of Gdańsk | 54°29′ N, 18°40′ E | 1997 | KC788204 |
| CCNP1402 |  | 54°30′ N, 18°33′ E | 2005 | KC751415 |
| CCNP1403 |  | 54°29′ N, 18°40′ E | 1997 | KC788205 |
| CCNP1423 |  |  | 2011 | no data |
| CCNP1424 |  |  | 2011 | no data |
| CCNP1425 |  |  | 2011 | no data |
| CCNP1426 |  | 54°35′ N, 18°47′ E | 2012 | KX889405 |
| CCNP1427 |  | 54°35′ N, 18°47′ E | 2012 | KX889406 |
| CCNP1428 |  | 54°30′ N, 18°33′ E | 2012 | KX889407 |
| CCNP1430 |  | 54°35′ N, 18°47′ E | 2012 | KX889408 |
| CCNP1431 |  | 54°26′ N, 18°34′ E | 2012 | KX889410 |
| CCNP1436 |  | 54°35′ N, 18°47′ E | 2012 | KX889409 |
| CCNP1440 |  | 54°30′ N, 18°33′ E | 2013 | KX889411 |
| CCY9414 | Bornholm Basin |  | 1988 | KX889413 |
| BY1 | Arkona Basin |  | 1986 | KX889412 |

**Table S2.** Peptides detected in the Baltic *Nodularia spumigena* strains. Abbreviations stand for: AcSer-acetylserine, Agm-agmatine, Bu-butanoic acid, Hex-hexanoic acid, Hpla-hydroxyphenyl lactic acid, Hph-homophenylalanine, Hty-homotyrosine, MePro-metylproline, MetO-methionine sufloxide, Met(O_2_)-methionine sulfone, MeHty-methylhomotyrosine, Oct-octanoic acid. Novel peptide structures are denoted with an asterisk.

| ***m*/*z*** | **Peptide Structure** |
| --- | --- |
| **Spumigins** | |
| 655 | (Hpla + 42) + Hty + Pro + Arg |
| 653 | (Hpla + 42) + Hty + MePro + Argal |
| 641 | (Hpla + 42) + Hty + Pro + Argol |
| 639 | (Hpla + 42) + Hty + Pro + Argal |
| 613a | Hpla + Hty + MePro + Argol |
| 613b | Hpla + Hty + Pro + Arg |
| 611 | Hpla + Hty + MePro + Argal |
| 599 | Hpla + Hty + Pro + Argol |
| 597 | Hpla + Hty + ProZ + Argal |
| 595 | Hpla + Hph + MePro + Argal |
| 583a | Hpla + Tyr + Pro + Argal |
| 583b | Hpla + Hty + MePro + Agm |
| 581 | Hpla + Hph + Pro + rgal |
| 575 | (Hpla + 42) + Leu + Pro + Argal |
| **Aeruginosins** | |
| 615 | Oct + Tyr + Choi + Argal |
| 603 | Hex + Tyr + Choi + Arg |
| 589 | Hex + Tyr + Choi + Argol |
| 587 | Hex + Tyr + Choi + Argal |
| 559 | Bu + Tyr + Choi + Agm |
| **Pseudoaeruginosins** | |
| 533 | Hex + Tyr + MePro + Argol |
| 531 | Hex + Tyr + Choi + Argal |
| **Nodularins** | |
| 825 | Cyclo[MeAsp + Arg + Adda + Glu + Mdhb] |
| 811 | Cyclo[Asp + Arg + Adda + Glu + Mdhb] |
| **Anabaenopeptins** | |
| 934 | Phe + CO + [Lys + Val + Hty + MeHty + MetO] |
| 932 | Ile + CO + [Lys + MetO + Hph + MeHty + MetO] |
| 930 | Ile + CO + [Lys + MetO_2_ + Hph + MeHty + AcSer] |
| 918 | Phe + CO + [Lys + Val + Hph + MeHty + MetO] |
| 916a | Ile + CO + [Lys + MetO + Hph + MeHty + Met] |
| 916b | Phe + CO + [Lys + Val + Hty + MeHty + AcSer] |
| 914 | Ile + CO + [Lys + MetO + Hph + MeHty + AcSer] |
| 902 | Phe + CO + [Lys + Val + Hph + MeHty + Met] |
| 900a | Phe + CO + [Lys + Val + Hph + MeHty + AcSer] |
| 900b | Ile + CO + [Lys + Met + Hph + MeHty + Met] |
| 898a | Ile + CO + [Lys + Met + Hph + MeHty + AcSer] |
| 898b | Ile + CO + [Lys + MetO + Hph + MeHph + AcSer] |
| *886 | Phe + CO + [Lys + Val + Hph + MeHph + Met] |
| 884a | Ile + CO + [Lys + Met + Hph + MeHph + Met] |
| 884b | Phe + CO + [Lys + Val + Hph + MeHph + AcSer] |
| 884c | Ile + CO + [Lys + Val + Hph + MeHty + MetO] |
| 882a | Ile + CO + [Lys + Met + Hph + MeHph + AcSer] |
| 882b | Ile + CO + [Lys + Ile + Hph + MeHty + Met] |
| 880 | Ile + CO + [Lys + Ile + Hph + MeHty + AcSer] |
| 872 | Ile + CO + [Lys + MetO + Hph + MeHty + Ser] |
| 868 | Ile + CO + [Lys + Val + Hph + MeHty + Met] |
| 866 | Ile + CO + [Lys + Val + Hph + MeHty + AcSer] |
| *864 | Ile + CO + [Lys + Ile + Hph + MeHph + AcSer] |
| 858 | Phe + CO + [Lys + Val + Hph + MeHtyr + Ser] |
| 856a | Ile + CO + [Lys + Met + Hph + MeHty + Ser] |
| 856b | Ile + CO + [Lys + MetO + Hph + MeHph + Ser] |
| *852 | Ile + CO + [Lys + Val + Hph + MeHph + Met] |
| *850 | Ile + CO + [Lys + Val + Hph + MeHph + AcSer] |
| 842 | Phe + CO + [Lys + Ile + Hty + MeAla + Phe] |
| *840 | Ile + CO + [Lys + Met + Hph + MeHph + Ser] |
| 828 | Phe + CO + [Lys + Val + Hty + MeAla + Phe] |
| 824 | Ile + CO + [Lys + Val + Hph + MeHty + Ser] |
| *822 | Ile + CO + [Lys + Ile + Hph + MeHph + Ser] |
| *808a | Ile + CO + [Lys + Val + Hph + MeHph + Ser] |
| 808b | Ile + CO + [Lys + Ile + Hty + MeAla + Phe] |
| 794 | Ile + CO + [Lys + Val + Hty + MeAla + Phe] |
| **Cyanopeptolin-like** | |
| *848 | ?[? + Ahp + Phe + MeHty + ?] |

**Table S3.** Peptides produced by the Baltic *Nodularia spumigena* strains. Two chemotype clusters were identified CT_A and CT_B. In CT_B, different sub-groups were found CT_B1, CT_B2 and CT_B3. In the table, the peptides are characterized by *m*/*z* values of their molecular ions. New peptide structures are denoted with an asterisk

| ***m*/*z*** | **Peptide Symbol** | **CT_A** | | | | | | | | **CT_B** | | | | | | | | | | | | | | | | | ***m*/*z*** |
| --- | --- | --- | --- | --- | --- | --- | --- | --- | --- | --- | --- | --- | --- | --- | --- | --- | --- | --- | --- | --- | --- | --- | --- | --- | --- | --- | --- |
|  |  |  |  |  |  |  |  |  |  | **CT_B1** | | **CT_B2** | | | | | | **CT_B3** | | | | | | | | |  |
|  |  | **CCNP 1402** | **BY 1** | **CCY 9414** | **AV 1** | **CCNP 1427** | **CCNP 1436** | **KAC 7** | **KAC 71** | **KAC 70** | **KAC 87** | **CCNP 1401** | **CCNP 1403** | **KAC 11** | **CCNP 1426** | **CCNP 1430** | **KAC 13** | **CCNP 1440** | **CCNP 1431** | **CCNP 1428** | **CCNP 1423** | **CCNP 1424** | **CCNP 1425** | **KAC 66** | **KAC 64** | **KAC 68** |  |
| **SPUMIGINS** | | | | | | | | | | | | | | | | | | | | | | | | | | | |
| 655 | SPU 654 |  |  |  |  |  |  |  |  |  |  | X | X | X |  |  |  |  |  |  |  |  |  |  |  |  | 655 |
| 653 | SPU 652 |  |  |  |  |  |  |  |  |  |  | X | X | X |  |  |  |  |  |  |  |  |  |  |  |  | 653 |
| 641 | SPU 640 |  |  |  |  |  |  |  |  |  |  | X | X | X |  |  |  |  |  |  |  |  |  |  |  |  | 641 |
| 639 | SPU 638 |  |  |  |  |  |  |  |  |  |  | X | X | X |  |  |  |  |  |  |  |  |  |  |  |  | 639 |
| 613a | SPU A | X | X | X | X | X | X | X | X | X | X | X | X |  | X | X | X | X | X | X |  |  |  | X | X | X | 613a |
| 613b | SPU C |  |  |  |  |  |  |  |  | X |  | X | X | X |  | X | X | X |  |  |  |  |  | X | X | X | 613b |
| 611 | SPU E | X | X | X | X | X | X | X | X | X | X | X | X | X | X | X | X | X | X | X | X | X | X | X | X | X | 611 |
| 599 | SPU D | X | X | X | X | X | X | X | X | X | X | X | X | X | X | X | X | X | X | X | X | X | X | X | X | X | 599 |
| 597 | SPU F | X | X | X | X | X | X | X | X | X | X | X | X | X | X | X | X | X | X | X | X | X | X | X | X | X | 597 |
| 595 | SPU G | X | X | X | X | X | X | X | X | X | X |  |  | X | X |  | X | X | X | X | X | X | X | X | X | X | 595 |
| 583a | SPU 582a |  |  |  |  |  |  |  |  |  |  | X | X |  |  |  |  |  |  |  |  |  |  |  |  |  | 583a |
| 583b | SPU 582b |  |  |  | X |  |  |  |  |  |  |  |  |  |  |  |  |  |  |  |  |  |  |  |  |  | 583b |
| 581 | SPU H | X | X | X | X |  | X | X | X | X | X |  |  |  | X | X |  |  | X |  |  |  |  | X | X | X | 581 |
| 575 | SPU 574 |  |  |  |  |  |  |  |  |  |  | X | X |  |  |  |  |  |  |  |  |  |  |  |  |  | 575 |
| **AERUGINOSINS** | | | | | | | | | | | | | | | | | | | | | | | | | | | |
| 615 | NAL 3 | X | X | X | X |  |  | X | X |  |  | X | X | X |  |  |  |  |  |  |  |  |  | X | X | X | 615 |
| 603 | AER 602 | X | X | X | X |  |  | X | X |  | X | X | X | X |  |  |  |  |  |  |  |  |  | X | X | X | 603 |
| 589 | NOL 3 | X | X | X | X |  |  | X | X | X | X | X | X | X |  |  |  |  |  |  |  |  |  | X | X | X | 589 |
| 587 | NAL 2 | X | X | X | X |  |  | X | X | X | X | X | X | X |  |  |  | X |  |  | X | X | X | X | X | X | 587 |
| 559 | NAL 1 |  |  | X | X |  |  | X | X |  |  |  |  | X |  |  |  |  |  |  |  |  |  | X | X | X | 559 |
| **PSEUDOAERUGINOSINS** | | | | | | | | | | | | | | | | | | | | | | | | | | | |
| 531 | NS 1 | X | X | X | X |  |  |  | X |  | X |  |  |  |  |  |  |  |  |  |  |  |  |  |  |  | 531 |
| 533 | NS 2 | X | X | X | X |  |  |  | X |  | X |  |  |  |  |  |  |  |  |  |  |  |  |  |  |  | 533 |
| **NODULARINS** | | | | | | | | | | | | | | | | | | | | | | | | | | | |
| 825 | NOD | X | X | X | X | X | X | X | X | X | X | X | X | X | X | X | X | X | X | X | X | X | X | X | X | X | 825 |
| 811 | [dMeAsp^3^] NOD | X | X | X | X | X | X | X | X | X | X | X | X | X | X | X | X | X | X | X | X | X | X | X | X | X | 811 |
| **ANABAENOPEPTINS** | | | | | | | | | | | | | | | | | | | | | | | | | | | |
| 934 | NP 933 |  |  |  |  |  |  |  |  |  |  |  |  |  |  |  |  | X | X | X | X | X | X | X | X | X | 934 |
| 932 | NP 931 | X | X | X | X | X | X | X | X |  |  |  |  |  |  |  |  |  |  |  |  |  |  |  |  |  | 932 |
| *m*/*z* | Peptide symbol | **CT_A** | | | | | | | | **CT_B** | | | | | | | | | | | | | | | | | ***m*/*z*** |
|  |  |  |  |  |  |  |  |  |  | **CT_B1** | | **CT_B2** | | | | | | **CT_B3** | | | | | | | | |  |
|  |  | **CCN 1402** | **BY 1** | **CCY 9414** | **AV 1** | **CCNP 1427** | **CCNP 1436** | **KAC 7** | **KAC 71** | **KAC 70** | **KAC 87** | **CCNP 1401** | **CCNP 1403** | **KAC 11** | **CCNP 1426** | **CCNP 1430** | **KAC 13** | **CCNP 1440** | **CCNP 1431** | **CCNP 1428** | **CCNP 1423** | **CCNP 1424** | **CCNP 1425** | **KAC 66** | **KAC 64** | **KAC 68** |  |
| 930 | NP A | X |  | X | X |  |  |  | X |  |  |  |  |  |  |  |  |  |  |  |  |  |  |  |  |  | 930 |
| 918 | NP 917 |  |  |  |  |  |  |  |  |  |  |  |  |  |  |  |  | X | X | X | X | X | X | X | X | X | 918 |
| 916a | NP 915a | X | X | X | X | X | X | X | X |  |  |  |  |  |  |  |  |  |  |  |  |  |  |  |  |  | 916a |
| 916b | NP 915b |  |  |  |  |  |  |  |  |  |  |  |  |  |  |  |  | X | X | X | X | X | X |  |  |  | 916b |
| 914 | NP B | X | X | X | X | X | X | X | X |  |  |  |  |  |  |  |  |  |  |  |  |  |  |  |  |  | 914 |
| 902 | NP 901 |  |  |  |  |  |  |  |  |  |  |  |  |  |  |  |  | X | X | X | X | X | X | X | X | X | 902 |
| 900a | NP 899 |  |  |  |  |  |  |  |  |  |  |  |  |  |  |  |  | X | X | X | X | X | X | X | X | X | 900a |
| 900b | [Met^6^]  NP C | X | X | X | X | X | X | X | X |  |  |  |  |  |  |  |  |  |  |  |  |  |  |  |  |  | 900b |
| 898a | NP C | X | X | X | X | X | X | X | X |  |  |  |  |  |  |  |  |  |  |  |  |  |  |  |  |  | 898a |
| 898b | [MeHph^5^] NP B | X | X | X | X | X | X | X | X |  |  |  |  |  |  |  |  |  |  |  |  |  |  |  |  |  | 898b |
| *886 | NP 885 |  |  |  |  |  |  |  |  |  |  |  |  |  |  |  |  | X | X |  |  |  |  | X | X | X | 886 |
| 884a | NP 883a | X | X | X | X | X | X | X | X |  |  |  |  |  |  |  |  |  |  |  |  |  |  |  |  |  | 884a |
| 884b | NP 883b |  |  |  |  |  |  |  |  |  |  |  |  |  |  |  |  | X | X | X | X | X | X | X | X | X | 884b |
| 884c | NP 883c |  |  |  |  |  |  |  |  | X | X |  |  |  |  |  |  |  |  |  |  |  |  |  |  |  | 884c |
| 882a | NP 881a | X | X | X | X | X | X | X | X |  |  |  |  |  |  |  |  |  |  |  |  |  |  |  |  |  | 882a |
| 882b | NP 881b | X | X | X | X | X | X | X | X |  |  |  |  |  |  |  |  |  |  |  |  |  |  |  |  |  | 882b |
| 880 | NP 879 | X | X | X | X | X | X | X | X |  |  |  |  |  |  |  |  |  |  |  |  |  |  |  |  |  | 880 |
| 872 | [Ser^6^] NP B | X | X | X | X | X | X | X | X |  |  |  |  |  |  |  |  |  |  |  |  |  |  |  |  |  | 872 |
| 868 | NP 867 |  |  |  | X |  |  |  |  | X | X |  |  |  |  |  |  |  |  |  |  |  |  |  |  |  | 868 |
| 866 | NP 865 |  |  |  | X |  |  |  |  | X | X |  |  |  |  |  |  |  |  |  |  |  |  |  |  |  | 866 |
| *864 | NP 863 |  |  |  | X |  |  |  |  |  |  |  |  |  |  |  |  |  |  |  |  |  |  |  |  |  | 864 |
| 858 | AP 857 |  |  |  |  |  |  |  |  |  |  |  |  |  |  |  |  |  |  |  |  |  |  | X |  | X | 858 |
| 856a | NP 855a | X | X | X | X | X | X | X | X |  |  |  |  |  |  |  |  |  |  |  |  |  |  |  |  |  | 856a |
| 856b | NP 855b | X | X | X |  | X | X | X | X |  |  |  |  |  |  |  |  |  |  |  |  |  |  |  |  |  | 856b |
| *852 | NP 851 |  |  |  |  |  |  |  |  | X | X |  |  |  |  |  |  |  |  |  |  |  |  |  |  |  | 852 |
| *850 | NP 849 |  |  |  | X |  |  |  |  |  | X |  |  |  |  |  |  |  |  |  |  |  |  |  |  |  | 850 |
| 842 | AP 841 |  |  |  |  |  |  |  |  |  |  | X | X |  | X | X | X |  |  |  |  |  |  |  |  |  | 842 |
| *840 | NP 839 | X | X | X | X | X | X | X | X |  |  |  |  |  |  |  |  |  |  |  |  |  |  |  |  |  | 840 |
| 828 | AP D |  |  |  |  |  |  |  |  |  |  | X | X |  | X | X | X |  |  |  |  |  |  |  |  |  | 828 |
| 824 | NP 823 |  |  |  |  |  |  |  | X | X | X |  |  |  |  |  |  |  |  |  |  |  |  |  |  |  | 824 |
| *822 | NP 821 | X | X |  | X | X | X | X | X |  |  |  |  |  |  |  |  |  |  |  |  |  |  |  |  |  | 822 |
| *808a | NP 807 |  |  |  |  |  |  |  |  | X | X |  |  |  |  |  |  |  |  |  |  |  |  |  |  |  | 808a |
| 808b | AP 807 |  |  |  |  |  |  |  |  |  |  |  |  | X |  |  |  |  |  |  |  |  |  |  |  |  | 808b |
| 794 | AP J |  |  |  |  |  |  |  |  |  |  |  |  | X |  |  |  |  |  |  |  |  |  |  |  |  | 794 |
| **CYANOPEPTOLIN–LIKE PEPTIDE** | | | | | | | | | | | | | | | | | | | | | | | | | | | |
| *848 |  |  |  |  | X |  |  |  |  |  |  |  |  |  |  |  |  |  |  |  |  |  |  |  |  |  | 848 |

**Table S4**. Relative intensity of extracted ion peaks of spumigins produced by the Baltic *Nodularia spumigena* strains. The type of residue in position 3 (Pro/MePro) is indicated. Spumigins detected in trace amounts, at ion intensity lower than 10^5^ are marked with letter t.

| **Chemotype Cluster** | ***N. spumigena* Strain** | **[M + H]**  **[*m*/*z*]** | **Pro^3^/MePro^3^** | **Ion Intensity [10−⁷]** |
| --- | --- | --- | --- | --- |
| **CT_A** | CCNP1402 | 613 | MePro | 2.3 |
|  |  | 611 | MePro | 4.0 |
|  |  | 599 | Pro | 1.4 |
|  |  | 597 | Pro | t |
|  |  | 595 | MePro | 2.4 |
|  |  | 581 | Pro | t |
|  | BY1 | 613 | MePro | 1.5 |
|  |  | 611 | MePro | t |
|  |  | 599 | Pro | 2.2 |
|  |  | 597 | Pro | t |
|  |  | 595 | MePro | t |
|  |  | 581 | Pro | t |
|  | CCY9414 | 613 | MePro | 4.5 |
|  |  | 611 | MePro | 4.5 |
|  |  | 599 | Pro | t |
|  |  | 597 | Pro | t |
|  |  | 595 | MePro | 2.7 |
|  |  | 581 | Pro | t |
|  | AV1 | 613 | MePro | 5.0 |
|  |  | 611 | MePro | 5.4 |
|  |  | 599 | Pro | 5.4 |
|  |  | 597 | Pro | 4.5 |
|  |  | 595 | MePro | 5.0 |
|  |  | 583 | MePro | t |
|  |  | 581 | Pro | 3.3 |
|  | CCNP1427 | 613 | MePro | t |
|  |  | 611 | MePro | 3.8 |
|  |  | 599 | Pro | t |
|  |  | 597 | Pro | t |
|  |  | 595 | MePro | 1.5 |
|  | CCNP1436 | 613 | MePro | 1.2 |
|  |  | 611 | MePro | 4.0 |
|  |  | 599 | Pro | 0.5 |
|  |  | 597 | Pro | 4.0 |
|  |  | 595 | MePro | 0.8 |
|  |  | 581 | Pro | t |
|  | KAC7 | 613 | MePro | 3.5 |
|  |  | 611 | MePro | 2.5 |
|  |  | 599 | Pro | t |
|  |  | 597 | Pro | t |
|  |  | 595 | MePro | 0.6 |
|  |  | 581 | Pro | t |
|  | KAC71 | 613 | MePro | 5.4 |
|  |  | 611 | MePro | 5.8 |
|  |  | 599 | Pro | t |
|  |  | 597 | Pro | 4.5 |
|  |  | 595 | MePro | 5.0 |
|  |  | 581 | Pro | t |
| **CT_B1** | KAC70 | 613 | MePro | t |
|  |  | 613 | Pro | t |
|  |  | 611 | MePro | 1.6 |
|  |  | 599 | Pro | t |
|  |  | 597 | Pro | t |
|  |  | 595 | MePro | t |
|  |  | 581 | Pro | t |
|  | KAC87 | 613 | MePro | t |
|  |  | 611 | MePro | 4.9 |
|  |  | 599 | Pro | t |
|  |  | 597 | Pro | 4.0 |
|  |  | 595 | MePro | 5.7 |
|  |  | 581 | Pro | t |
| **CT_B2** | CCNP1401 | 655 | Pro | t |
|  |  | 653 | MePro | t |
|  |  | 641 | Pro | t |
|  |  | 639 | Pro | t |
|  |  | 613 | MePro | t |
|  |  | 613 | Pro | t |
|  |  | 611 | MePro | t |
|  |  | 599 | Pro | 2.5 |
|  |  | 597 | Pro | 1.3 |
|  |  | 583 | Pro | t |
|  |  | 575 | Pro | t |
|  | CCNP1403 | 655 | Pro | t |
|  |  | 653 | MePro | t |
|  |  | 641 | Pro | t |
|  |  | 639 | Pro | t |
|  |  | 613 | MePro | t |
|  |  | 613 | Pro | t |
|  |  | 611 | MePro | t |
|  |  | 599 | Pro | t |
|  |  | 597 | Pro | 2.5 |
|  |  | 583 | Pro | t |
|  |  | 575 | Pro | t |
|  | KAC11 | 655 | Pro | t |
|  |  | 653 | MePro | t |
|  |  | 641 | Pro | t |
|  |  | 639 | Pro | t |
|  |  | 613 | Pro | t |
|  |  | 611 | MePro | t |
|  |  | 599 | Pro | 2.2 |
|  |  | 597 | Pro | 2.7 |
|  |  | 595 | MePro | t |
|  | CCNP1426 | 613 | MePro | t |
|  |  | 611 | MePro | 4.5 |
|  |  | 599 | Pro | t |
|  |  | 597 | Pro | t |
|  |  | 595 | MePro | t |
|  |  | 581 | Pro | t |
|  | CCNP1430 | 613 | MePro | t |
|  |  | 613 | Pro | t |
|  |  | 611 | MePro | 1.4 |
|  |  | 599 | Pro | 2.4 |
|  |  | 597 | Pro | 1.8 |
|  |  | 581 | Pro | t |
|  | KAC13 | 613 | MePro | 4.5 |
|  |  | 613 | Pro | t |
|  |  | 611 | MePro | 3.5 |
|  |  | 599 | Pro | t |
|  |  | 597 | Pro | t |
|  |  | 595 | MePro | t |
| **CT_B3** | CCNP1440 | 613 | MePro | 2.2 |
|  |  | 613 | Pro | t |
|  |  | 611 | MePro | 2.5 |
|  |  | 599 | Pro | 4.5 |
|  |  | 597 | Pro | 2.0 |
|  |  | 595 | MePro | t |
|  | CCNP1431 | 613 | MePro | 2.5 |
|  |  | 611 | MePro | 5.4 |
|  |  | 599 | Pro | 2.0 |
|  |  | 597 | Pro | 4.0 |
|  |  | 595 | MePro | t |
|  |  | 581 | Pro | t |
|  | CCNP1428 | 613 | MePro | t |
|  |  | 611 | MePro | t |
|  |  | 599 | Pro | t |
|  |  | 597 | Pro | t |
|  |  | 595 | MePro | t |
|  | CCNP1423 | 611 | MePro | t |
|  |  | 599 | Pro | t |
|  |  | 597 | Pro | t |
|  |  | 595 | MePro | t |
|  | CCNP1424 | 611 | MePro | t |
|  |  | 599 | Pro | t |
|  |  | 597 | Pro | t |
|  |  | 595 | MePro | t |
|  | CCNP1425 | 611 | MePro | t |
|  |  | 599 | Pro | t |
|  |  | 597 | Pro | t |
|  |  | 595 | MePro | t |
|  | KAC66 | 613 | MePro | t |
|  |  | 613 | Pro | t |
|  |  | 611 | MePro | t |
|  |  | 599 | Pro | 4.0 |
|  |  | 597 | Pro | 5.0 |
|  |  | 595 | MePro | t |
|  |  | 581 | Pro | t |
|  | KAC64 | 613 | MePro | t |
|  |  | 613 | Pro | t |
|  |  | 611 | MePro | t |
|  |  | 599 | Pro | 5.5 |
|  |  | 597 | Pro | 4.4 |
|  |  | 595 | MePro | t |
|  |  | 581 | Pro | 1.5 |
|  | KAC68 | 613 | MePro | 2.9 |
|  |  | 613 | Pro | t |
|  |  | 611 | MePro | t |
|  |  | 599 | Pro | 4.5 |
|  |  | 597 | Pro | 5.0 |
|  |  | 595 | MePro | t |
|  |  | 581 | Pro | 5.6 |
